# A new species of the *Aenictus wroughtonii* group (Hymenoptera, Formicidae) from South-East China

**DOI:** 10.3897/zookeys.391.7213

**Published:** 2014-03-20

**Authors:** Michael Staab

**Affiliations:** 1 Chair of Nature Conservation and Landscape Ecology, Institute of Earth and Environmental Sciences, University of Freiburg, Tennenbacherstraße 4, 79106 Freiburg, Germany

**Keywords:** Aenictinae, *Aenictus gutianshanensis*, army ants, species description, taxonomy

## Abstract

A new species of army ant from the *Aenictus wroughtonii* group is described and illustrated based on the worker caste. *Aenictus gutianshanensis* Staab, **sp. n.** is known form a single colony collected in the subtropical mixed evergreen broad-leaved forest of the Gutianshan National Nature Reserve, South-East China. The new species is probably most closely related to *A. vieti* Jaitrong & Yamane, 2010 known from North Vietnam and Taiwan. It is suggested that the abundant records of *A. camposi* Wheeler & Chapman, 1925 from East and South-East China should be reevaluated, as they are probably *A. gutianshanensis* or *A. vieti* and not *A. camposi*, which is distributed in Sundaland, the Philippines, and the southernmost part of continental South-East Asia.

## Introduction

The genus *Aenictus*, which is the only genus of the dorylomorph subfamily Aenictinae, is the largest genus of army ants. Army ants are characterized by several specialized morphological, behavioral, and ecological adaptations, such as a nomadic life style, highly specialized mating systems, and mass raiding for arthropod prey (Schneirla and Reyes 1966, Gotwald 1995, Kronauer 2009). *Aenictus* is restricted to the tropical and subtropical regions of the Old World and Australia (Gotwald 1995). Almost all *Aenictus* species are specialized predators of other ants (e.g. Rościszewski and Maschwitz 1994, Hirosawa et al. 2000, Jaitrong and Yamane 2011), however they can also supplement their diet with plant-based resources (Staab in press).

Until now, 179 species (9 synonyms, 2 unavailable) and 30 subspecies (13 synonyms, 2 unavailable) have been validly described (AntCat 2014). Recently, Jaitrong and coworkers established 12 species groups based on the worker caste (Jaitrong and Yamane 2011), and comprehensively revised the Oriental and Australasian *Aenictus* fauna (Jaitrong and Yamane 2010, Jaitrong et al. 2010, Jaitrong and Yamane 2011, Wiwatwitaya and Jaitrong 2011, Jaitrong and Hashimoto 2012, Jaitrong and Yamane 2012, Jaitrong and Wiwatwitaya 2013, Jaitrong and Yamane 2013).

The *Aenictus wroughtonii* species group has been revised in detail by Jaitrong et al. (2010). The group contains seven species in the Oriental and Australasian faunal region that can easily be separated from other conspecific *Aenictus* species by the combination of a yellowish and slender body, very long antennal scapes, and a rounded anterior clypeal margin bearing several denticles (Jaitrong et al. 2010, Jaitrong and Yamane 2011). In the present paper, a new species of the *Aenictus wroughtonii* group from South-East China is described as new to science based on the worker caste.

## Methods

All morphological observations were made with a Leica SD6 stereomicroscope. Measurements were taken with an ocular micrometer. Images were produced using a Keyence VHX2000 (Osaka, Japan) digital microscope.

The general worker terminology follows Jaitrong et al. (2010) and Jaitrong and Yamane (2011).

All measurements are expressed in millimeters. Abbreviations used for measurements and indices follow Jaitrong et al. (2010) and Jaitrong and Yamane (2011) and are:

CI Cephalic index, HW / HL × 100.

HL Maximum head length in full-face view, measured from the anterior clypeal margin (excluding the projecting clypeal teeth) to the midpoint of a line drawn across the posterior margin of the head.

HW Maximum head width in full face view.

ML Mesosomal length measured from the point at which the pronotum meets the cervical shield to the posterior base of the metapleuron in profile.

MTL Maximum length of mid tibia, excluding the proximal part of the articulation which is received into the distal end of the femur.

PL Petiole length measured from the anterior margin of the peduncle to the posteriormost point of tergite.

SI Scape index, SL / HW × 100.

SL Scape length excluding the basal constriction and condylar bulb.

TL Total length, measured roughly from the anterior margin of head to the tip of gaster in stretched specimens.

### Depositories of type material

CASC California Academy of Science Collection, San Francisco, California, USA.

IZAS Insect Collection of the Institute of Zoology, Chinese Academy of Sciences, Beijing, China.

ZMBH Museum für Naturkunde, Berlin, Germany.

## Results

### *Aenictus wroughtonii* species group

Jaitrong et al. (2010) and Jaitrong and Yamane (2011) defined this species group as follows:

*Head narrow; occipital margin lacking collar. Antenna long, consisting of 10 segments, with a strikingly long scape attaining or extending beyond posterolateral corner of head (but in one Vietnamese species the scape shorter, not reaching posterolateral corner of head). Anterior clypeal margin roundly convex with 5–10 denticles. Mandible triangular, with masticatory margin bearing 8–12 minute inconspicuous denticles in addition to large apical tooth with a sharp apex; basal margin of mandible lacking denticles. Frontal carina short; parafrontal ridge feeble and incomplete. Mesosoma narrow and elongate. Legs very slender. Subpetiolar process weakly developed or almost absent. Head and gaster entirely smooth and shiny. Nearly entire body clear yellow to yellowish brown; typhlatta spot absent*.

#### Aenictus gutianshanensis

Staab sp. n.

http://zoobank.org/F14B8EED-1D2E-4931-A0AA-F3697502BEEF

http://species-id.net/wiki/Aenictus_gutianshanensis

Figs 1 –5

##### Holotype.

Worker from China, Zhejiang Province, Gutianshan National Nature Reserve, ca. 30 km NW of Kaihua, 29°12'54"N, 118°7'18"E, ca. 250 m above sea level, 28.VI.2009, leg. Andreas Schuldt, label: “CSP26/SW7(2009)”, deposited in IZAS.

**Figures 1–4.**
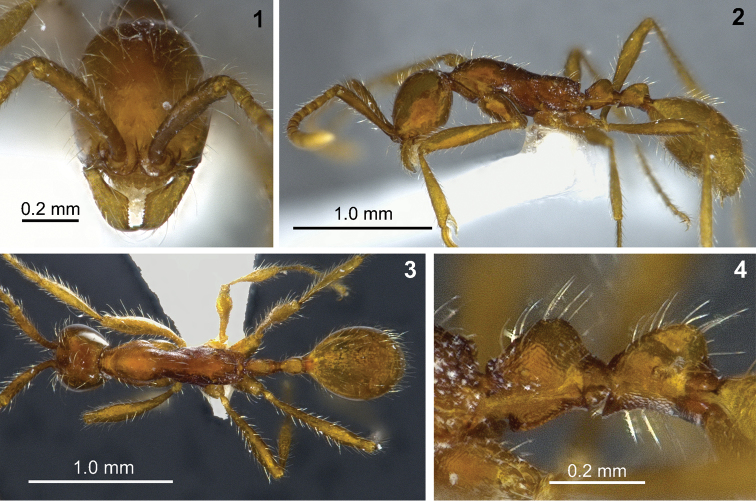
*Aenictus gutianshanensis* sp. n. (holotype). **1** Head in full-face view **2** body in profile **3** body in dorsal view **4** propodeal junction petiole and postpetiole in profile.

**Figure 5.**
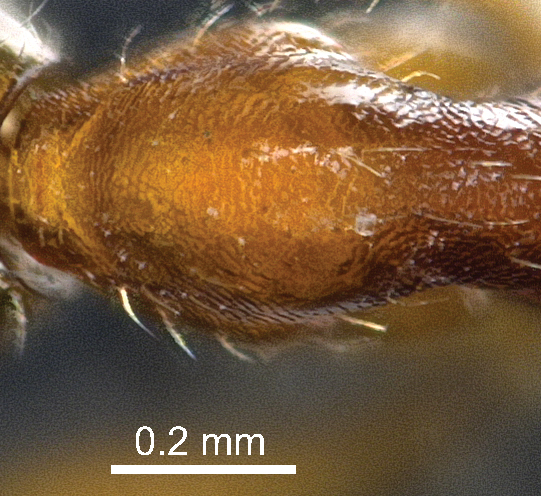
*Aenictus gutianshanensis* sp. n. (holotype), sculpture of pronotal dorsum.

##### Paratypes.

Five workers, same data as holotype. Three deposited in IZAS; one each deposited in ZMBH and CASC. All type specimens were collected in a single pitfall trap in a secondary mixed evergreen broad-leaved forest.

##### Measurements and indices.

**Holotype:** TL 3.30, HL 0.68, HW 0.63, SL 0.70, ML 1.17, MTL 0.75, PL 0.30, CI 93, SI 112.

**Paratypes** (n=5): TL 3.10–3.30, HL 0.69–0.75, HW 0.60–0.65, SL 0.65–0.70, ML 1.17–1.25, MTL 0.69–0.83, PL 0.29–0.31, CI 87–91, SI 104–113.

##### Worker description.

Head in full-face view elliptical, slightly longer than broad, with convex sides and almost straight posterior margin of head. Antennal scape long, reaching posterior corner of head; antennal segments II-X each longer than broad; II as long as III, but longer than each of IV-VII; terminal segment (X) longer than each of II-IX; the last four segments forming an indistinct club. Frontal carina long, extending slightly beyond the posterior margin of antennal torulus. Clypeus short with its anterior margin slightly convex, bearing 7-8 bluntly rounded denticles. Mandible subtriangular, masticatory margin straight, with a large curved apical tooth which is followed by 9-10 minutes teeth on masticatory margin. With mesosoma in profile, pronotum dorsally convex, not distinctly separated from mesonotum by a promesonotal suture. Propodeum slightly lower than promesonotum, its dorsal outline gently sloping posteriorly; propodeal junction angulate; declivity of propodeum straight in the dorsal part, concave in the ventral part when viewed in profile, encircled by a thin rim. Petiole in profile as long as high, its node convex dorsally. Subpetiolar process present, its ventral margin almost straight, bearing a thin rim below, anteroventral corner angulate. Postpetiole slightly longer than petiole, its node convex dorsally in profile; ventral postpetiolar process developed, angulate, bearing a thin rim below, slightly projecting over the posterior part of the petiole.

Head including mandible smooth and shiny; antennal scape punctate. Entire mesosoma finely reticulate, dorsal face of pronotum finely reticulate but shiny, reticulation on mesopleuron, metapleuron and lateral face of propodeum finer than on pronotum, appearing almost punctate in magnification lower 64×. Entire petiole finely reticulate. Postpetiole finely reticulate, except the dorsum smooth and shiny. Gaster smooth and shiny. Coxae finely reticulate, femora densely punctate, tibiae sparsely punctate.

Body except anterior part of mesonotum with abundant standing hairs and interdispersed short hairs; length of longest hairs on dorsa of head and pronotum 0.20–0.30 mm. Antennal scape and legs with abundant standing hairs. Head, mandible, gaster and legs yellowish brown. Mesosoma, antennal scape, petiole and postpetiole reddish brown.

Male and female are unknown.

##### Etymology.

**Figure 6.**
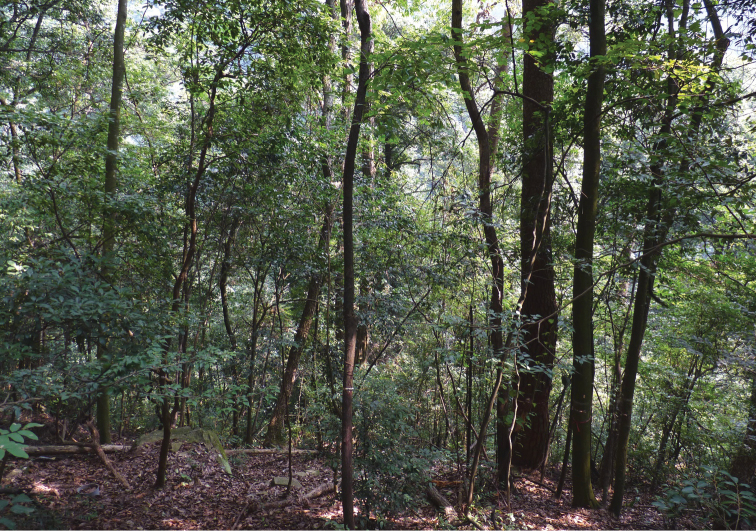
Typical mixed evergreen broad-leaved forest at the type locality, the Gutianshan National Nature Reserve.

The scientific name is after the type locality, the Gutianshan National Nature Reserve (Fig. 6) in South-East China.

##### Distribution.

South-East China; only known from the type series.

##### Ecology.

No direct biological information is available. The type series was collected in a single pitfall trap in a secondary mixed evergreen broad-leaved forest. Thus, the species probably lives and forages on and in the leaf-litter preying on small ants of the subfamily Formicinae, as it has been previously reported for species in the *Aenictus wroughtonii* group (Rościszewski and Maschwitz 1994, Jaitrong et al. 2010). Possible prey species of the genera *Prenolepis* and *Nylanderia* are common at the type locality (M. Staab, unpublished data).

## Discussion

*Aenictus* is one of the most species-rich ant genera in China and worldwide (Guénard and Dunn 2012, AntCat 2014), and the present paper raises the number of described species to 180. However, several *Aenictus* species have been described based only on males. As males of *Aenictus gutianshanensis* and many other *Aenictus* species are so far unknown, further collections supported by genetic work are needed in the future to clarify the relationship between male and worker based species names. Recently, the exhaustive reviews of Jaitrong and coworkers (Jaitrong and Yamane 2010, Jaitrong et al. 2010, Jaitrong and Yamane 2011, Wiwatwitaya and Jaitrong 2011, Jaitrong and Hashimoto 2012, Jaitrong and Yamane 2012, Jaitrong and Wiwatwitaya 2013, Jaitrong and Yamane 2013) provided detailed taxonomic and biogeographic information for the Oriental *Aenictus* fauna. Nevertheless, additional new species from the region have been described by various authors since these revisions (e.g. Jaitrong and Nur-Zati 2010, Bharti et al. 2012). Due to their highly specialized colony cycle which is characterized by reproduction through colony fission only (Gotwald 1995), most *Aenictus* species have a low dispersal potential and rather small and limited distribution ranges (e.g. Jaitrong et al. 2010, 2012, Jaitrong and Yamane 2013).

*Aenictus gutianshanensis* can be easily distinguished from all other species of the *Aenictus wroughtonii* group by the pronotum, the petiole, and the side of the postpetiole completely finely reticulate (see Jaitrong et al. 2010 for detailed species descriptions including a key to the *Aenictus wroughtonii* group; the same key is available online at http://www.antwiki.org/wiki/Key_to_Aenictus_wroughtonii_group_species). The new species is most similar to *Aenictus vieti* Jaitrong & Yamane, 2010 and to *Aenictus camposi* Wheeler & Chapman, 1925 but slightly larger in all measurements. In addition to having the pronotum and petiole completely finely reticulate, *Aenictus gutianshanensis* can be easily distinguished from *Aenictus vieti* and *Aenictus camposi* by the following characters (characters for *Aenictus vieti* and *Aenictus camposi* are given in brackets, see Jaitrong et al. 2010): ventral margin of subpetiolar process almost straight (ventral margin convex), femora densely punctate (smooth and shiny in Jaitrong et al. 2010, but superficially and irregularly sculptured and shiny in two paratypes examined by the reviewer), postpetiolar process more developed with a rim below (less developed, without ventral rim), and longest standing hairs on pronotal dorsum distinctly longer (maximal 0.13 mm).

In China, *Aenictus camposi* has been recorded from several provinces in East and South-East China. (Guénard and Dunn 2012). However, *Aenictus camposi* is a clearly tropical species whose distribution is restricted to Sundaland, the Philippines, and the southernmost part of continental South-East Asia (Jaitrong et al. 2010). Jaitrong and Yamane (in Jaitrong et al. 2010) described *Aenictus vieti* from Taiwan and North Vietnam. The authors recommended treating the Chinese records of *Aenictus camposi* as *Aenictus vieti*, which is morphologically similar. However, *Aenictus vieti* has not yet been recorded from mainland China. Thus, I also recommend reevaluating the Chinese *Aenictus camposi* for their identity. The type locality of *Aenictus gutianshanensis* is in the Zhejiang Province, and at least *Aenictus camposi* specimens collected further north, e.g. in Anhui or Hubei, may be *Aenictus gutianshanensis*.

There are probably several *Aenictus* species which still await discovery and description in the tropical and subtropical forests of the Oriental region. However, these forests are under high land-use pressure and are increasingly being cleared for agriculture (Gibbs et al. 2010, Miettinen et al. 2011, Hansen et al. 2013). As top predators, *Aenictus* and other army ants are sensitive to the negative effects of forest fragmentation and anthropogenic land use (Matsumoto et al. 2009). *Aenictus gutianshanensis* was discovered in South-East China, a region that was once covered by species rich subtropical forests which have largely been converted to timber plantations and to agricultural land (López-Pujol et al. 2006). The type locality, the Gutianshan National Nature Reserve (Bruelheide et al. 2011), despite being secondary forest, is one of the larger fragments of the historically wide ranging mixed evergreen broad-leaved forest in South-East China. These areas, as well as other secondary forest fragments in China and elsewhere probably contain several new ant species waiting to be discovered.

## Supplementary Material

XML Treatment for Aenictus gutianshanensis

## References

[B1] AntCat. Available from: http://www.antcat.org/catalog/429477 [accessed 3 March 2014]

[B2] Bharti H Wachkoo AA Kumar R (2012) Two remarkable new species of *Aenictus* (Hymenoptera: Formicidae) from India. Journal of Asia-Pacific Entomology 15: 291-294. 10.1016/j.aspen.2012.02.002

[B3] Bruelheide H Böhnke M Both S Fang T Assmann T Baruffol M Bauhus J Buscot F Chen XY Ding BY Durka W Erfmeier A Fischer M Geissler C Guo DL Guo LD Härdtle W He JS Hector A Kröber W Kühn P Lang AC Nadrowski K Pei KQ Scherer-Lorenzen M Shi XZ Scholten T Schuldt A Trogisch S von Oheimb G Welk E Wirth C Wu YT Yang XF Zeng XQ Zhang SR Zhou HZ Ma KP Schmid B (2011) Community assembly during secondary forest succession in a Chinese subtropical forest. Ecological Monographs 81: 25-41. 10.1890/09-2172.1

[B4] Gibbs HK Ruesch AS Achard F Clayton MK Holmgren P Ramankutty N Foley JA (2010) Tropical forests were the primary sources of new agricultural land in the 1980s and 1990s. Proceedings of the National Academy of Sciences of the United States of America 107: 16732-16737. 10.1073/pnas.0910275107 20807750 10.1073/pnas.0910275107 PMC2944736

[B5] Gotwald WHJ (1995) Army ants: the biology of social predation. Cornell University Press, Ithaca, NY, U.S.A. 320 pp.

[B6] Guénard B Dunn RR (2012) A checklist of the ants of China. Zootaxa 3558: 1-77

[B7] Hansen MC Potapov PV Moore R Hancher M Turubanova SA Tyukavina A Thau D Stehman SV Goetz SJ Loveland TR Kommareddy A Egorov A Chini L Justice CO Townshend JRG (2013) High-resolution global maps of 21st-century forest cover change. Science 342: 850-853. 10.1126/science.1244693 24233722 10.1126/science.1244693

[B8] Hirosawa H Higashi S Maryati M (2000) Food habits of *Aenictus* army ants and their effects on the ant community in a rain forest of Borneo. Insectes Sociaux 47: 42-49. 10.1007/s000400050007

[B9] Jaitrong W Hashimoto Y (2012) Revision of the *Aenictus minutulus* species group (Hymenoptera: Formicidae: Aenictinae) from Southeast Asia. Zootaxa 3426: 29-44

[B10] Jaitrong W Nur-Zati MA (2010) A new species of the ant genus *Aenictus* (Hymenoptera: Formicidae: Aenictinae) from the Malay Peninsula. Sociobiology 56: 449-454

[B11] Jaitrong W Wiwatwitaya D (2013) Two new species of the *Aenictus pachycerus* species group (Hymenoptera: Formicidae: Aenictinae) from Southeast Asia. Raffles Bulletin of Zoology 61: 97-102

[B12] Jaitrong W Yamane S (2010) The army ant *Aenictus silvestrii* and its related species in Southeast Asia, with a description of a new species (Hymenoptera: Formicidae: Aenictinae). Entomological Science 13: 328-333. 10.1111/j.1479-8298.2010.00385.x

[B13] Jaitrong W Yamane S (2011) Synopsis of *Aenictus* species groups and revision of the *A. currax* and *A. laeviceps* groups in the eastern Oriental, Indo-Australian, and Australasian regions (Hymenoptera: Formicidae: Aenictinae). Zootaxa 3128: 1-46

[B14] Jaitrong W Yamane S (2012) Review of the Southeast Asian species of the *Aenictus javanus* and *Aenictus philippinensis* species groups (Hymenoptera, Formicidae, Aenictinae). Zookeys 193: 49-78. 10.3897/zookeys.193.2768 22679379 10.3897/zookeys.193.2768 PMC3361139

[B15] Jaitrong W Yamane S (2013) The *Aenictus ceylonicus* species group (Hymenoptera, Formicidae, Aenictinae) from Southeast Asia. Journal of Hymenoptera Research 31: 165-233. 10.3897/jhr.31.4274

[B16] Jaitrong W Yamane S Tasen W (2012) A sibling species of *Aenictus dentatus* FOREL, 1911 (Hymenoptera: Formicidae) from continental Southeast Asia. Myrmecological News 16: 133-138

[B17] Jaitrong W Yamane S Wiwatwitaya D (2010) The army ant *Aenictus wroughtonii* (Hymenoptera, Formicidae, Aenictinae) and related species in the oriental region, with descriptions of two new species. Japanese Journal of Systematic Entomology 16: 33-46

[B18] Kronauer DJC (2009) Recent advances in army ant biology (Hymenoptera: Formicidae). Myrmecological News 12: 51-65

[B19] López-Pujol J Zhang F-M Ge S (2006) Plant biodiversity in China: richly varied, endangered, and in need of conservation. Biodiversity and Conservation 15: 3983-4026. 10.1007/s10531-005-3015-2

[B20] Matsumoto T Itioka T Yamane S Momose K (2009) Traditional land use associated with swidden agriculture changes encounter rates of the top predator, the army ant, in Southeast Asian tropical rain forests. Biodiversity and Conservation 18: 3139-3151. 10.1007/s10531-009-9632-4

[B21] Miettinen J Shi C Liew SC (2011) Deforestation rates in insular Southeast Asia between 2000 and 2010. Global Change Biology 17: 2261-2270. 10.1111/j.1365-2486.2011.02398.x

[B22] Rościszewski M Maschwitz U (1994) Prey specialization of army ants of the genus *Aenictus* in Malaysia. Andrias 13: 179-187

[B23] Schneirla TC Reyes AY (1966) Raiding and related behaviour in two surface-adapted species of the Old World Doryline ant, *Aenictus*. Animal Behavior 14: 132-148. 10.1016/S0003-3472(66)80022-2 10.1016/s0003-3472(66)80022-2 5918237

[B24] Staab M (in press) The first observation of honeydew foraging in army ants since 1933: *Aenictus hodgsoni* Forel, 1901 tending *Eutrichosiphum heterotrichum* (Raychaudhuri, 1956) in South-East China. Asian Myrmecology.

[B25] Wiwatwitaya D Jaitrong W (2011) The army ant *Aenictus hottai* (Hymenoptera: Formicidae: Aenictinae) and related species in Southeast Asia, with a description of a new species. Sociobiology 58: 557-565

[B26] Wheeler WM Chapman JW (1925) The ants of the Philippine Islands. Part I, Dorylinae and Ponerinae. Philippine Journal of Science 28: 27-73

